# Contribution of Thyrotropin-Releasing Hormone to Cerebellar Long-Term Depression and Motor Learning

**DOI:** 10.3389/fncel.2018.00490

**Published:** 2018-12-12

**Authors:** Masashi Watanave, Yasunori Matsuzaki, Yasuyo Nakajima, Atsushi Ozawa, Masanobu Yamada, Hirokazu Hirai

**Affiliations:** ^1^Department of Neurophysiology and Neural Repair, Gunma University Graduate School of Medicine, Maebashi, Japan; ^2^Department of Medicine and Molecular Science, Gunma University Graduate School of Medicine, Maebashi, Japan; ^3^Research Program for Neural Signalling, Division of Endocrinology, Metabolism and Signal Research, Gunma University Initiative for Advanced Research, Maebashi, Japan

**Keywords:** thyrotropin-releasing hormone, motor learning, cerebellum, LTD, NO

## Abstract

Thyrotropin-releasing hormone (TRH) regulates various physiological activities through activation of receptors expressed in a broad range of cells in the central nervous system. The cerebellum expresses TRH receptors in granule cells and molecular layer interneurons. However, the function of TRH in the cerebellum remains to be clarified. Here, using TRH knockout (KO) mice we studied the role of TRH in the cerebellum. Immunohistochemistry showed no gross morphological differences between KO mice and wild-type (WT) littermates in the cerebellum. In the rotarod test, the initial performance of KO mice was comparable to that of WT littermates, but the learning speed of KO mice was significantly lower than that of WT littermates, suggesting impaired motor learning. The motor learning deficit in KO mice was rescued by intraperitoneal injection of TRH. Electrophysiology revealed absence of long-term depression (LTD) at parallel fiber-Purkinje cell synapses in KO mice, which was rescued by bath-application of TRH. TRH was shown to increase cyclic guanosine monophosphate (cGMP) content in the cerebellum. Since nitric oxide (NO) stimulates cGMP synthesis in the cerebellum, we examined whether NO-cGMP pathway was involved in TRH-mediated LTD rescue in KO mice. Pharmacological blockade of NO synthase and subsequent cGMP production prevented TRH-induced LTD expression in KO mice, whereas increase in cGMP signal in Purkinje cells by 8-bromoguanosine cyclic 3’,5’-monophosphate, a membrane-permeable cGMP analog, restored LTD without TRH application. These results suggest that TRH is involved in cerebellar LTD presumably by upregulating the basal cGMP level in Purkinje cells, and, consequently, in motor learning.

## Introduction

The tripeptideamide pyroGlu-His-Pro-NH2 was originally isolated from mammalian hypothalami, and is released from the hypothalamus. It stimulates the synthesis and secretion of pituitary thyrotropin. Thus, this small peptide was named thyrotropin-releasing hormone (TRH). However, it became evident that the biological action of TRH extends far beyond the thyroid hormone-associated pathway. TRH and TRH receptors are expressed in a wide range of cells in the central nervous system and are implicated in the regulation of various physiological activities including arousal, circadian rhythmicity, pain perception, and spinal motor function (Nillni and Sevarino, 1999; Yamada et al., 2003).

The cerebellum is a center of motor coordination and motor learning (Bellebaum and Daum, 2007; Peterburs and Desmond, 2016), and its impairment results in cerebellar ataxia and motor learning deficits (Bastian, 2011). Spinocerebellar degeneration is a major disorder that progressively affects cerebellar function. Its main symptoms include dysmetria, intention tremor, and dysarthria (Paulson et al., 2017). Currently, no fundamental cure has been identified for this disease. A synthetic TRH analog, taltirelin, was shown to ameliorate ataxic behavior in the hereditary rolling mouse Nagoya, a murine model of spinocerebellar ataxia (Nakamura et al., 2005). It is, thus, used mostly in Japan for the treatment of spinocerebellar degeneration. However, the mechanisms by which TRH and taltirelin mitigate cerebellar ataxia have not yet been fully clarified.

One known finding about an effect of TRH on the cerebellum is an increase in cyclic guanosine monophosphate (cGMP) content, following the systemic administration, without significant changes in other brain regions (Mailman et al., 1979). The cGMP content in the cerebellum is extremely high (about 10-fold than that in other brain regions) (Steiner et al., 1972). Previous studies have suggested primary presence of cGMP and cGMP-dependent protein kinase G (PKG) in Purkinje cells (PCs) (Mao et al., 1975; Schlichter et al., 1980), suggesting that cGMP and PKG play critical roles in PC function. Since the TRH receptor (subtype 2) is expressed in granule cells and molecular layer interneurons, but not in PCs (Heuer et al., 2000), it is assumed that TRH signals are first activated in granule cells and molecular layer interneurons, and then, transferred transsynaptically to PCs to increase the cGMP level. However, considering broad physiological activities of TRH in other brain regions (Yarbrough, 2003; Zhang and Van Den Pol, 2012; Zhang et al., 2013), TRH could have more divergent roles in the cerebellum. In this study, we examined TRH knockout (KO) mice to investigate the physiological role of TRH in cerebellar function.

## Materials and Methods

### Animals

We used 10- to 16-week-old TRH KO mice, which were generated previously (Yamada et al., 1997). All procedures for the care and treatment of animals were performed according to the Japanese Act on the Welfare and Management of Animals, and the Guidelines for Proper Conduct of Animal Experiments as issued by the Science Council of Japan. The experimental protocol was approved by the Institutional Committee of Gunma University (No. 17-026; 17-034). All efforts were made to minimize suffering and to reduce the number of animals that were used.

### Rotarod Test

The motor control ability of mice was evaluated using a rotarod test (MK-610A/RKZ; Muromachi Kikai, Tokyo, Japan). Mice were subjected to four trials separated by 30-min intervals on the rod while accelerating from 5 to 50 rpm in 5 min. For rescue experiments of motor ability, saline or TRH (Sigma Aldrich, St. Louis, MO, United States) was administered by intraperitoneal injection (30 mg/kg body weight [BW]) to KO mice 10 min before the first trial of the rotarod test.

### Immunohistochemistry

Mice were deeply anesthetized and perfused intracardially with 4% paraformaldehyde in 0.1 M phosphate buffer (PB). The entire brain was removed and immersed in 4% paraformaldehyde in 0.1 M PB for 8 h at 4°C. Parasagittal cerebellar slices (50 μm thickness) were prepared using a vibratome (VT1200S; Leica, Wetzlar, Germany). The tissue slices were permeabilized and blocked with 0.1 M PB containing fivefold-diluted G-Block (GenoStaff, Tokyo, Japan), 0.5% (w/v) Triton X-100, and 0.025% NaN_3_ (blocking solution). They were then incubated in blocking solution containing the following antibodies overnight at room temperature (26°C): rabbit monoclonal anti-calbindin D-28K (1:500; C2724; Merck, Darmstadt, Germany), mouse monoclonal anti-NeuN (1:1,000; MAB377; Merck), and goat polyclonal anti-parvalbumin (1:200; PV-Go-Af460; Frontier Institute, Hokkaido, Japan). After washing six times with 0.1 M PB, the slices were incubated for 4 h at room temperature (26°C) in the blocking solution containing the following secondary antibodies (1:1,000; all purchased from Thermo Fisher Scientific, Waltham, MA, United States): Alexa Fluor 488-conjugated donkey anti-rabbit immunoglobulin G, Alexa Fluor 568-conjugated donkey anti-mouse immunoglobulin G, and Alexa Fluor 647-conjugated donkey anti-goat immunoglobulin G. After washing six times with 0.1 M PB at RT, the slices were mounted on glass slides using ProLong Diamond Antifade Reagent (P36961; Thermo Fisher Scientific). Each specimen was observed using a fluorescent microscope (BZ-X700; Keyence, Osaka, Japan) or a confocal laser-scanning microscope (LSM 800; Carl Zeiss, Oberkochen, Germany).

**FIGURE 1:**
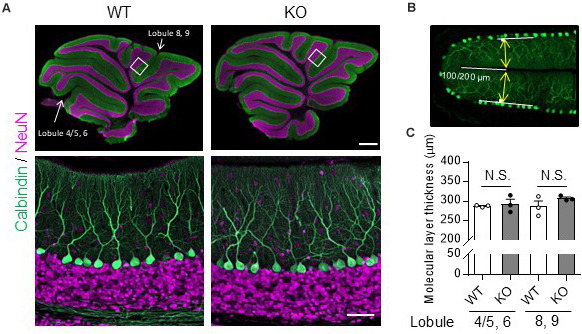
No obvious morphological differences in the cerebellum were found between TRH-KO mice and their WT littermates. Immunohistochemistry was used to compare sagittal cerebellar sections from TRH-KO mice to those from their WT littermates. Slices were double-immunostained for calbindin, a marker of PCs (green), and NeuN, a marker of granule cells (magenta). **(A)** Sagittal sections of the WT (left) and TRH-KO (right) cerebellum. The boxed areas in upper panels are enlarged. Scale bar: 500 μm (upper right) and 50 μm (lower right). KO, knock-out; WT, wild-type. **(B,C)** Quantitative analysis of the molecular layer thickness. The molecular layer thickness was measured at two different points on the sagittal section of the cerebellar vermis: lobule 4/5 and lobule 6 at 100 μm from the end of the primary fissure, and lobule 8 and lobule 9 at 200 μm from the end of the secondary fissure **(B)**. The molecular layer thickness of both sides of the fissure was measured, and the sum was defined as molecular layer thickness. There are no significant differences at both two points between genotypes **(C)**. KO, knock-out; N.S., not significant; WT, wild-type.

Molecular layer thickness was measured using ZEN 2 (blue edition) software (Carl Zeiss). Sagittal slices from the cerebellar vermis (±0.5 μm from the median) were immunostained with the anti-calbindin D-28K antibody and mounted. Twenty-four hours after the mounting, images were obtained from lobules 4/5 – 6 including the primary fissure and lobules 8 – 9 including the secondary fissure, using a confocal microscope (LSM800). The molecular layer thickness was measured 100 μm (primary) or 200 μm (secondary) from the inner end of the fissure (see Figure 1B). The molecular layer thickness of both sides of the fissure was measured using ZEN2, and the sum was defined as molecular layer thickness.

### Patch Clamp Recording

Parasagittal cerebellar slices (250 μm in thickness) were prepared from mice. Briefly, mice were anesthetized deeply by inhalation of isoflurane (3%) and killed by decapitation. The whole brain was quickly dissected out and immersed for a 2–3 min in an ice-cold solution containing the following (in mM): 234 sucrose, 26 NaHCO_3_, 2.5 KCl, 1.25 NaH_2_PO_4_, 11 glucose, 10 MgSO_4_, and 0.5 CaCl_2_ (pH 7.4 when bubbled with 95% O_2_ and 5% CO_2_). Parasagittal slices of the cerebellar vermis were obtained using a microslicer (ZERO1; Dosaka-EM, Kyoto, Japan). The slices were maintained in an extracellular solution containing (in mM): 125 NaCl, 2.5 KCl, 2 CaCl_2_, 1 MgCl_2_, 1.25 NaH_2_PO_4_, 26 NaHCO_3_, 10 D-glucose, and 0.1 picrotoxin, bubbled continuously with a mixture of 95% O_2_ and 5% CO_2_ at room temperature (26°C) for at least 1 h before commencing recording. PCs were visualized using a 40× water-immersion objective attached to an upright microscope (Axioskop, Carl Zeiss). Whole-cell recordings were made from PCs at room temperature (26°C) and the slices were continuously perfused with the extracellular solution during the experiment. The extracellular solution contained 0.1 mM picrotoxin, except for the solution used to record spontaneous and miniature inhibitory postsynaptic currents (sIPSCs and mIPSCs, respectively). The resistance of the patch pipette was 3–6 MΩ when filled with intracellular solution containing (in mM): 122.5 Cs methane sulfonate, 17.5 CsCl, 8 NaCl, 2 Mg adenosine triphosphate, 0.3 Na guanosine triphosphate, 10 4-(2-hydroxyethyl)-1-piperazineethanesulfonic acid, 0.2 ethylene glycol-bis(β-aminoethyl ether)-N,N,N’,N’-tetraacetic acid, and 5 sucrose (pH 7.2, adjusted with CsOH). The intracellular solution used for IPSC recording contained (in mM): 127.5 CsCl, 2 CaCl_2_, 1 MgCl_2_, 2 MgATP, 0.3 NaGTP, 10 HEPES (pH 7.3, adjusted with CsOH).

Stimulation pipettes were filled with the extracellular solution and placed in the molecular and granule layer to activate parallel fibers (PFs) and climbing fibers (CFs), respectively. PCs were clamped at -70 mV or -10 mV to record PF- or CF-evoked excitatory postsynaptic currents (EPSCs), respectively. Liquid junction potentials were not corrected. To estimate the passive electrical properties of the recorded PCs, we applied 10-mV hyperpolarizing voltage pulses (from -70 mV to -80 mV, 200 ms duration). The averaged trace of the 10 current responses was used for the estimation. Membrane capacitance and input resistance were calculated from the integral of the capacitive charging current, and from the steady-state current amplitude measured late during the pulse, respectively.

Selective stimulation of PFs and CFs was confirmed by paired-pulse facilitation (PPF) and paired-pulse depression (PPD) of EPSC amplitudes with a 50 ms inter-stimulus interval, respectively. To isolate metabotropic glutamate receptor type 1 (mGluR1)-mediated slow EPSCs, repetitive PF stimuli (100 Hz) were applied in the presence of 20 μM 2,3-dioxo-6-nitro-1,2,3,4-tetrahydrobenzo[f]quinoxaline-7-sulfonamide, which is a highly selective competitive antagonist of α-amino-3-hydroxy-5-methyl-4-isoxazolepropionic acid (AMPA)-type glutamate receptors (Libbey et al., 2016).

For analysis of long-term depression (LTD), PF EPSCs were monitored every 10 s. We always confirmed the stability of PF EPSCs for at least 10 min before LTD induction. Series resistance was continuously monitored every 10 s by applying small hyperpolarizing pulses. Data were discarded when the resistance values changed by >20% of the basal value during the course of the experiment. To induce LTD, we applied conjunctive stimulation consisting of 30 single PF stimuli paired with single 200-ms depolarizing pulses (-70 mV to +10 mV) repeated at 1 Hz. Amplitudes of PF EPSCs were averaged every minute (six traces) and normalized by the averaged value of the responses over 10 min immediately before the conjunctive stimulation. Some LTD recordings were made in the presence of 100 nM TRH (Sigma-Aldrich), 30 μM 8-bromoguanosine 3’,5’-cyclic monophosphate (8-bromo-cGMP) (Sigma-Aldrich) or 100 μM N^G^-Nitro-L-arginine methyl ester hydrochloride (L-NAME) (Dojindo Laboratory, Kumamoto, Japan) (Figures 5–7).

Inhibitory postsynaptic currents were recorded in the presence of 25 μM D-2-amino-5-phosphonopentanoic acid (D-APV) and 20 μM NBQX to block excitatory inputs. mIPSCs were recorded in the presence of 1 μM tetrodotoxin (TTX) to block action potentials, and events were detected using a semiautomatic event detector (Clampfit software, San Jose, CA, United States). mIPSC events whose were amplitudes less than 10 pA were discarded.

### Statistical Analysis

Significant differences were analyzed using Welch’s *t*-test and repeated-measures analysis of variance using GraphPad Prism 7 (GraphPad Software, San Diego, CA, United States). The data are expressed as the mean ± standard error of the mean.

## Results

### No Gross Morphological Changes in the Cerebellum in TRH-Deficient Mice

The cerebella of 14-week-old TRH-KO mice were compared morphologically to those of wild-type (WT) littermates. After perfusion fixation, the cerebella were cut into 50-μm-thick sections and double-immunostained for calbindin, a PC marker, and NeuN, a granule cell marker. The morphology of the cerebellar cortex was almost indistinguishable between the KO mice and WT littermates (Figure 1A). We measured molecular layer thickness quantitatively, and confirmed no significant differences between the KO mice and WT littermates (Figures 1B,C, Lobule 4/5, 6; *P* = 0.272, Lobule 8, 9; *P* = 0.728). Thus, TRH deficiency had no overt influence on cerebellar morphology.

### TRH-Deficient Mice Show Motor Learning Deficits

**FIGURE 2:**
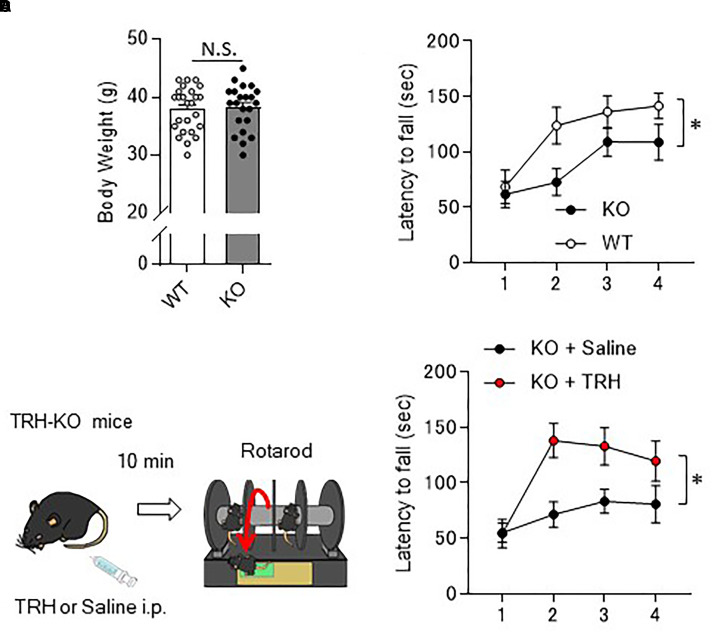
Motor learning deficits in TRH-KO mice and the restoration by exogenous TRH. **(A)** No difference in the body weights between genotypes. Body weight of 10- to 14-week-old TRH-KO mice and their WT littermates were measured [WT: *n* = 25 (male: 14, female: 12), KO: *n* = 22 (male: 12, female: 10)]. N.S., not significant. **(B)** Graph showing the averaged results of four rotarod trials [*n* = 12 (male: 8, female: 4) for both KO and WT mice], and the average time on the rod was plotted. KO, knock-out; WT, wild-type. **(C)** Schema showing the experimental procedure. Littermate TRH-KO mice received TRH (6 ml/kg BW, 5 mg/ml, *n* = 8 mice, male: 3, female: 5) or the same volume of saline (*n* = 8 mice, male: 3, female: 5) intraperitoneally (i.p.). The effects of the treatment were tested 10 min after the injection using the rotarod test. **(D)** Results of the rotarod test. Mice were subjected to four trials. ^∗^*P* < 0.05, as determined using repeated-measures analysis of variance. KO, knock-out; WT, wild-type.

As the behavior of TRH-KO mice in the home cages was indistinguishable from that of their WT littermates, we analyzed their motor ability more carefully using a rotarod apparatus. Numbers of males and females for the rotarod were adjusted between KO and WT mouse groups. However, since BW affects the rotarod performance, we compared the BW of 10- to 12-week-old KO mice with that of their WT littermates. The results showed no significant differences between genotypes (Figure 2A, *P* = 0.757). In the rotarod test, 10- to 14-week-old TRH-KO mice had similar performance to their WT littermates on the first trial. During the following trials, mice of both genotypes learned the task, and the time on the rod gradually increased. However, learning was significantly slower in the KO mice [Figure 2B, *F*(1,22) = 5.22, *P* = 0.032]. These results suggest that motor coordination was normal in TRH-KO mice, but that motor learning ability was significantly impaired.

### Rescue of Motor Learning Defect by Exogenous TRH

As revealed in the rotarod test, TRH-KO mice had impaired motor learning. This might be due to a developmental abnormality or merely the absence of TRH-triggered signaling. To address this issue, we administered TRH (6 ml/kg BW, 5 mg/ml) or similar volume of saline intraperitoneally to TRH-KO mice and assessed the effects of TRH on motor learning 10 min after the injection (Figure 2C). TRH-injected KO mice had significantly better performance than saline-injected KO mice in the rotarod test [Figure 2D, *F*(1,14) = 9.44, *P* = 0.008]. WT mice were treated similarly with TRH or saline, but, TRH, like saline, had no significant influence on the rotarod performance (Supplementary Figure S1). Notably, TRH-administered KO mice showed favorable rotarod performance, almost comparable to that of TRH-treated WT mice. These results suggest that the motor learning deficit was likely due to an absence of TRH signaling, which was restored by application of exogenous TRH.

### Normal Fast Synaptic Transmission at PF- and CF-PC Synapses in TRH-Deficient Mice

**FIGURE 3:**
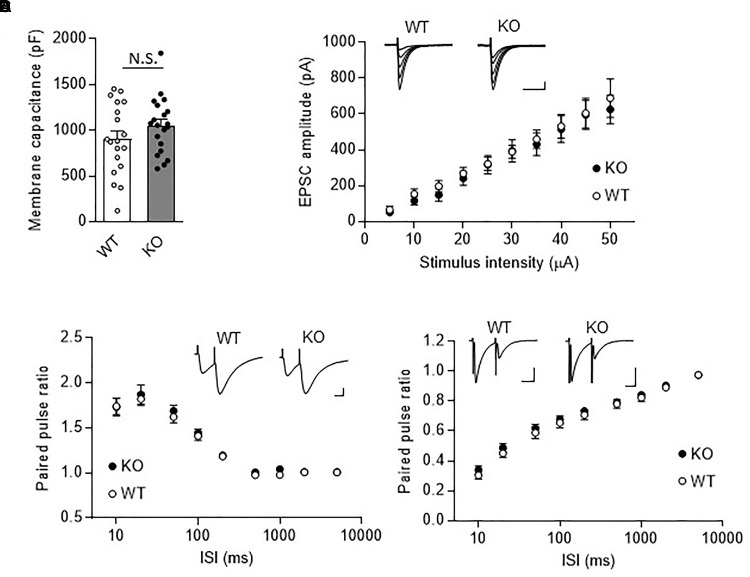
Normal fast synaptic transmission and short-term synaptic plasticity in TRH-KO mouse PCs. **(A)** Membrane capacitance in PCs from TRH-KO mice and their WT littermates (KO: 20 PCs from four mice, WT: 19 PCs from four mice). N.S., no significant difference as determined using Welch’s *t*-test. **(B)** Graph depicting the average sizes of PF EPSC amplitudes against the intensity of stimulus delivered to PFs (KO: 9 PCs from four mice, WT: 10 PCs from four mice). Representative traces of EPSCs at various stimulus intensities (5–50 μA) are presented above the graph. Scale bar: 100 pA, 50 ms. **(C,D)** Averaged graphs showing the PPF ratios of PF EPSCs **(C)** and the PPD ratios of CF EPSCs **(D)** determined based on the responses evoked by two stimuli with various interstimulus intervals. The PPF and PPD ratios were measured as the second EPSC amplitude normalized to the first EPSC amplitude. Representative traces for stimulation with a 20-ms interstimulus interval are presented above the graph. CF, climbing fiber; EPSC, excitatory postsynaptic current; KO, knock-out; PF, parallel fiber; PPD, paired-pulse depression; PPF, paired-pulse facilitation; WT, wild-type.

We next examined synaptic transmission at PF- and CF-PC synapses using the whole cell patch-clamp technique. Fast PF and CF EPSCs were elicited in PCs by electrical stimulation of PFs or CFs, respectively. Membrane capacitance (Figure 3A, KO: 1053.0 ± 68.5 pF, WT: 903.8 ± 88.9 pF, *P* = 0.19), and rise time and decay time constants of the fast PF and CF EPSCs (Table 1) were comparable between the KO mice and their WT littermates. We then examined the relationship between stimulus intensity and evoked PF EPSC amplitude, and found no difference between the KO mice and their WT littermates (Figure 3B). Moreover, there were no significant differences in short-term synaptic plasticity, as assessed based on the ratio of PPF in PF EPSCs and the ratio of PPD in CF EPSCs, between the KO mice and their WT littermates (Figures 3C,D).

**Table 1:** PF and CF EPSC kinetics were normal in TRH-KO mouse PCs.

	PF EPSC		CF EPSC	
	Rise (ms)	Decay (ms)	Rise (ms)	Decay (ms)
WT	2.59 ± 0.13	14.25 ± 1.15	0.65 ± 0.08	9.29 ± 1.04
KO	2.69 ± 0.16	16.25 ± 1.09	0.61 ± 0.05	8.40 ± 0.47
*P*-value	0.65	0.22	0.72	0.45


*Statistically significant differences were determined using Welch’s *t*-test. EPSC, excitatory postsynaptic current; KO, knock-out; PF, parallel fiber; WT, wild-type.*

### Absence of LTD at PF-PC Synapses in TRH-Deficient Mice

**FIGURE 4:**
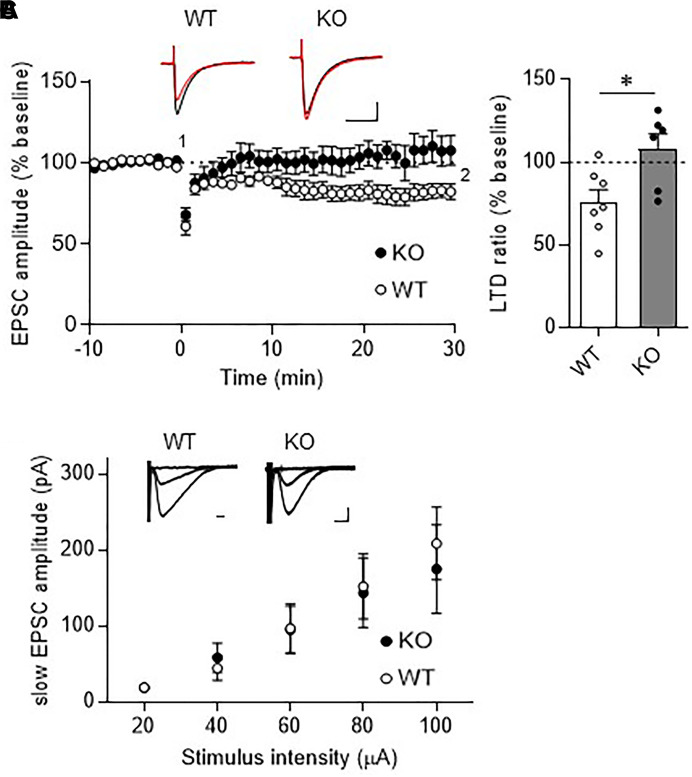
Absence of LTD in TRH-KO mice. **(A)** Time course depicting PF EPSC amplitudes before and after a conjunctive stimulation (KO: six PCs from four mice, WT: eight PCs from five mice). Representative traces before (1) and 30 min after (2) the conjunctive stimulation is shown as black and red lines. Scale bar: 100 pA, 50 ms. **(B)** Percent ratios of PF EPSC amplitudes 30 min after a conjunctive stimulation relative to those before the conjunctive stimulation. Asterisks indicate statistically significant differences as determined using Welch’s *t*-test (^∗^*P* < 0.05). **(C)** Input–output graph showing slow EPSC amplitudes against the intensity of stimulus delivered to PFs. Representative traces of slow EPSCs at 20 μA, 60 μA, and 100 μA stimulus intensity are presented above the graph. Scale bar: 100 pA, 200 ms (WT: eight PCs from four mice, KO: seven PCs from three mice). EPSC, excitatory postsynaptic current; KO, knock-out; LTD, long-term depression; PF, parallel fiber; WT, wild-type.

Long-term depression at PF-PC synapses is thought to be a cellular basis of motor learning. We thus examined whether LTD could be induced at PF-PC synapses in TRH-KO mice. After recording stable PF-EPSCs for at least 10 min, conjunctive stimulation (electrical stimulation to PFs combined with depolarization of the recording PC) induced LTD at PF-PC synapses in WT mice. However, similar stimulation failed to induce LTD in TRH-KO mice (Figures 4A,B, KO: 108.0 ± 9.3%, WT: 82.1 ± 4.5%, *P* = 0.039).

Repetitive PF stimulation leads to glutamate spillover from the synaptic clefts between the PF terminals and the dendritic spines of PCs. This in turn leads to activation of Gq/11 protein-coupled mGluR1, which is localized postsynaptically at perisynaptic sites of PC dendritic spines (Hirai and Kano, 2018). Activation of mGluR1 leads to production of diacylglycerol and inositol-triphosphate, the latter of which triggers calcium release from the endoplasmic reticulum. The elevated calcium together with diacylglycerol activates protein kinase C (PKC), which plays a key role in LTD induction (Matsuda et al., 2000; Hirai, 2001; Chung et al., 2003). Thus, activation of mGluR1 and downstream signaling is indispensable for LTD at these synapses. To confirm proper mGluR1 activation following repetitive PF stimulation in TRH-KO mouse PCs, we assessed the generation of slow EPSCs. PFs were stimulated with five pulses at 100 Hz in the presence of NBQX to block AMPA receptor-mediated fast EPSCs. The amplitude of the evoked slow EPSC was plotted against the increasing electrical stimulation (Figure 4C). There were no statistically significant differences in the slow EPSC amplitude between TRH-KO mice and their WT littermates. These results suggest that TRH deficiency had no influence on the activation of mGluR1 in PCs.

### Rescue of Cerebellar LTD Defects by Exogenous TRH

**FIGURE 5:**
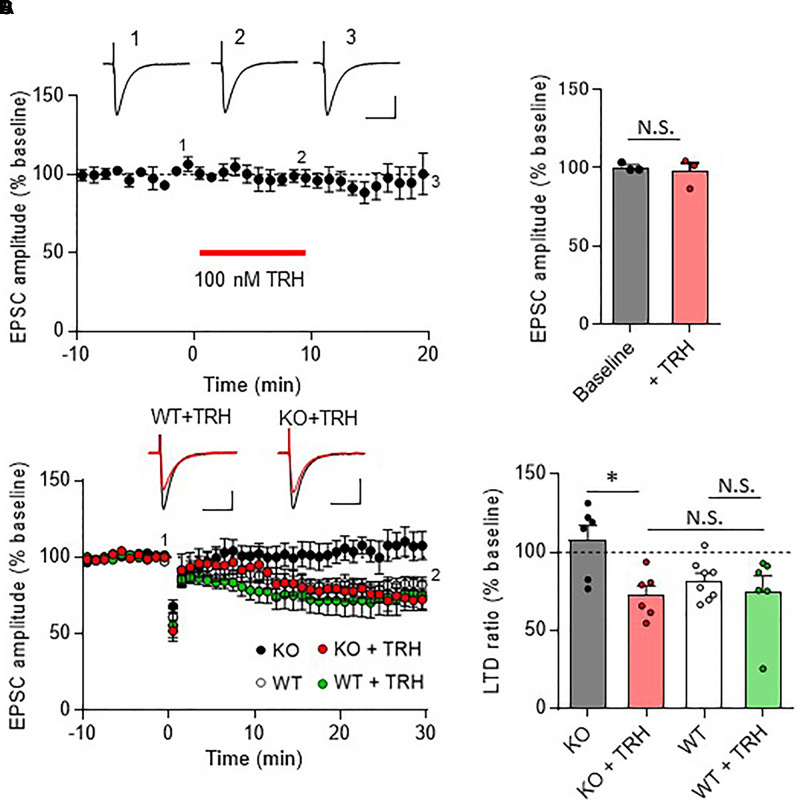
Rescue of cerebellar LTD in TRH-deficient mice following treatment with exogenous TRH. **(A)** No influence of TRH on the amplitudes of PF EPSCs elicited in TRH-KO mouse PCs. TRH (100 nM) was bath-applied to cerebellar slices for 10 min, as indicated by the red horizontal bar (*n* = 3 PCs from three TRH-KO mice). Representative traces before (1) and 10 min after (2) the TRH application and washout (3) are shown. Scale bar: 100 pA, 50 ms. **(B)** Summary graph showing the effects of TRH on EPSCs, which were calculated by dividing the averaged EPSC amplitude values obtained from –5 to 0 min or 5 to 10 min by those obtained from –10 to 0 min. **(C)** Cerebellar LTD in the presence of TRH (100 nM). Conjunctive stimulation used for LTD induction was performed at time 0 (KO: six PCs from four mice, WT: eight PCs from five mice, KO + TRH: six PCs from four mice, WT + TRH: six PCs from three mice). Representative traces before (1) and 30 min after (2) the conjunctive stimulation is shown as black and red lines, respectively. Scale bar: 100 pA, 50 ms. **(D)** Summary graph showing PF EPSC amplitudes 30 min after conjunctive stimulation relative to the baseline (100%). Data from the control KO mice and those from the control WT mice **(C,D)**, which are the same as those shown in other figures, are presented for comparison. ^∗^*P* < 0.05, as determined using Welch’s *t*-test. EPSC, excitatory postsynaptic current; KO, knock-out; LTD, long-term depression; N.S., not significant; PF, parallel fiber; WT, wild-type.

Next, we examined whether failure of LTD induction in TRH-KO mice could be restored by exogenous TRH. Bath application of TRH (100 μM) to cerebellar slices from TRH-KO mice did not influence the PF EPSC amplitude (Figures 5A,B). However, PF stimulation paired with depolarization of the PC recording effectively induced LTD (Figures 5C,D, KO + TRH: 72.7 ± 5.9%, *P* = 0.011). Similar bath application of TRH to cerebellar slices from WT mice had no significant effect on LTD expression and the degree of PF EPSC depression (Figures 5C,D, WT: 82.2 ± 4.5%, WT + TRH: 74.7 ± 10.3%, *P* = 0.528). These results suggest that absence of LTD in the TRH-KO mice were likely due to an absence of TRH signaling, which was restored by application of exogenous TRH.

### Involvement of NO-cGMP Pathway in TRH-Mediated Rescue of LTD

**FIGURE 6:**
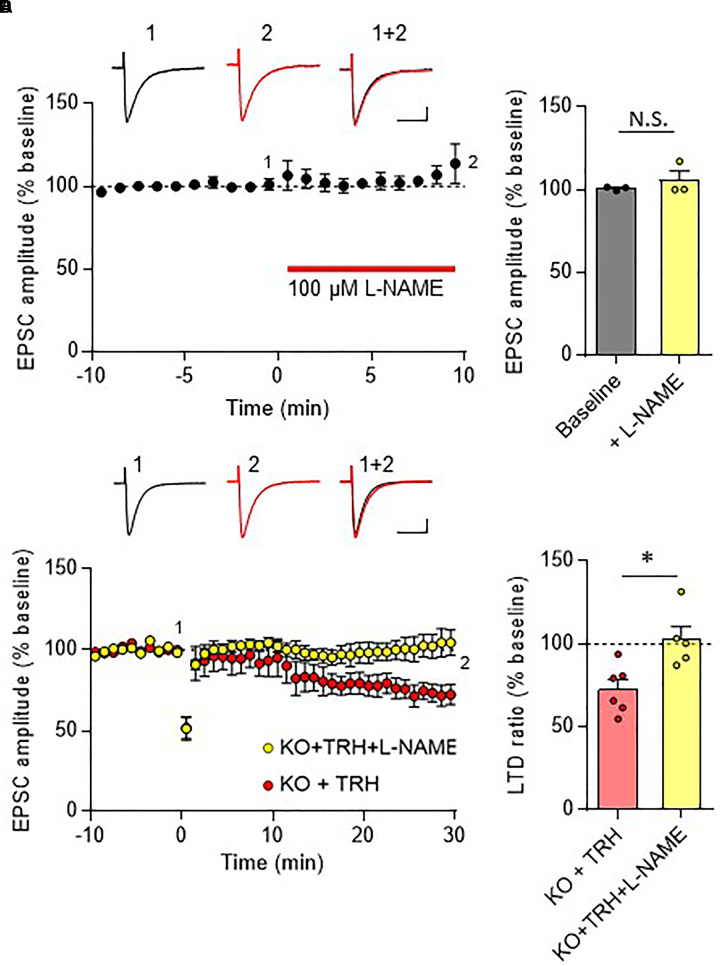
Inhibition of the NO synthase eliminates the TRH-mediated rescue of cerebellar LTD. **(A)** Bath application of L-NAME, a cell-permeable NO synthase inhibitor, had no influence on PF EPSC. Representative traces before (1) and 10 min after (2) the L-NAME treatment are shown as black and red lines, respectively. Superimposed images (1 + 2) are also shown. Scale bar: 100 pA, 50 ms. **(B)** Summary graph showing the effects of 8-bromo-cGMP on EPSCs, which were calculated by dividing the averaged EPSC amplitude values obtained from –5 to 0 min or 5 to 10 min by those obtained from –10 to 0 min. **(C)** TRH fails to induce LTD in PCs from KO mice in the presence of 100 μM L-NAME. Representative traces before (1) and 30 min after (2) the conjunctive stimulation is shown as black and red lines, respectively. Superimposed images (1 + 2) are also shown. Scale bar: 100 pA, 50 ms. **(D)** Summary graph showing depression ratios, which were calculated by dividing the averaged EPSC amplitude values obtained from 25 to 30 min by those obtained from –10 to 0 min (KO + TRH: six PCs from four mice, KO + TRH + L-NAME: five PCs from three mice). Data from KO mouse cerebellum treated only with TRH (KO + TRH) **(C,D)**, which are the same as those shown in the other figures, are presented for comparison. Asterisks indicate statistically significant differences, as determined using Welch’s *t*-test. ^∗^*P* < 0.05. EPSC, excitatory postsynaptic current; KO, knock-out; L-NAME, *N*-nitro-L-arginine methyl ester; LTD, long-term depression; PF, parallel fiber.

**FIGURE 7:**
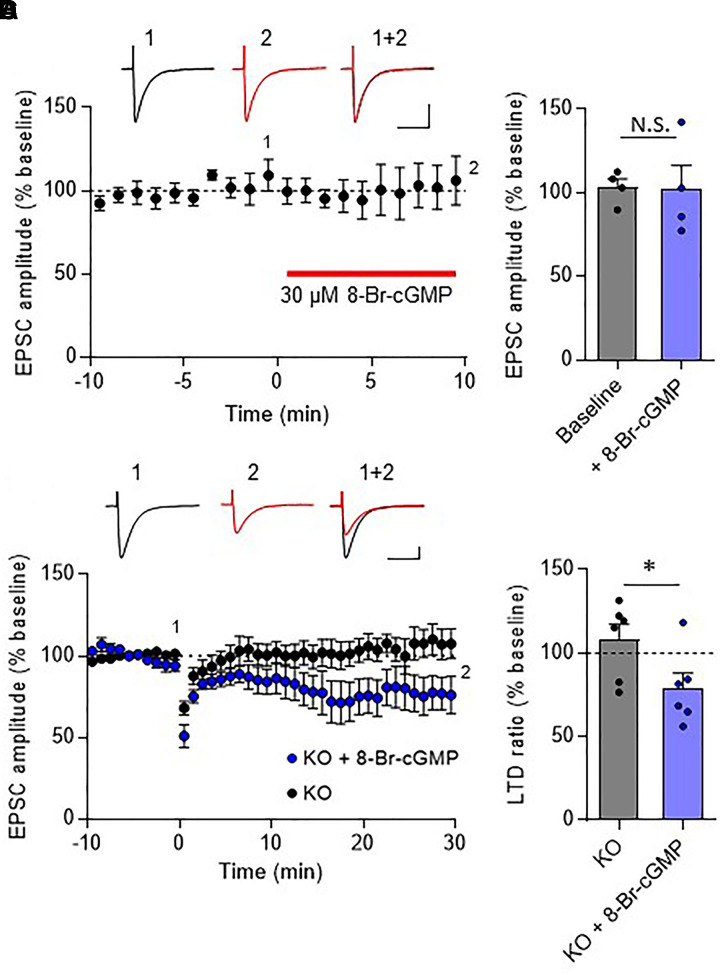
Rescue of cerebellar LTD in TRH-deficient mice by 8-bromo-cGMP treatment. **(A)** Effects of 8-bromo-cGMP on baseline PF EPSCs. Thirty micromolar 8-bromo-cGMP was bath-applied to cerebellar slices for 10 min, as indicated using the red horizontal bar (*n* = 4 PCs from three mice). Representative traces before (1) and 10 min after (2) the 8-bromo-cGMP treatment are shown as black and red lines, respectively. Superimposed images (1 + 2) are also shown. Scale bar: 100 pA, 50 ms. **(B)** Summary graph showing the effects of 8-bromo-cGMP on EPSCs, which were calculated by dividing the averaged EPSC amplitude values obtained from –5 to 0 min or 5 to 10 min by those obtained from –10 to 0 min. **(C)** Robust LTD in PCs from KO mice in the presence of 30 μM 8-bromo-cGMP. Representative traces before (1) and 30 min after (2) the conjunctive stimulation is shown as black and red lines, respectively. Superimposed images (1 + 2) are also shown. Scale bar: 100 pA, 50 ms. **(D)** Summary graph showing depression ratios, which were calculated by dividing the averaged EPSC amplitude values obtained from 25 to 30 min by those obtained from –10 to 0 min (KO: six PCs from four mice, KO + 8-bromo-cGMP: six PCs from three mice). Data from control KO mice **(C,D)**, which are the same as those shown in the other figures, are presented for comparison. Asterisks indicate statistically significant differences, as determined using Welch’s *t*-test. ^∗^*P* < 0.05. EPSC, excitatory postsynaptic current; KO, knock-out; LTD, long-term depression; PF, parallel fiber.

In the cerebellar cortex, TRH receptors (TRH-R2) are expressed in granule cells and molecular layer interneurons, but not in PCs (Heuer et al., 2000), while TRH was shown to increase cGMP in PCs (Mao et al., 1975; Mailman et al., 1979; Schlichter et al., 1980). NO is a possible mediator of the trans-synaptic event. NO is synthesized by neuronal NO synthase, which is selectively expressed in granule cells and molecular layer interneurons (Vincent and Kimura, 1992; Rodrigo et al., 1994; Schilling et al., 1994; Ihara et al., 2006). Thus, it is assumed that NO, which is produced in these cells upon TRH receptor activation, diffuses into dendritic spines of PCs, and activates a guanylyl cyclase to synthesize cGMP. However, considering the highly bioactive nature of TRH, different mechanisms may contribute to the rescue of cerebellar LTD by exogenous TRH in TRH-KO mice. To clarify this, TRH was perfused to cerebellar slices, with NO production blocked by L-NAME, a cell-permeable NO synthase blocker. Bath application of L-NAME (100 μM) did not affect the amplitude of PF EPSC (Figures 6A,B). However, TRH-mediated rescue of LTD was completely blocked by L-NAME (Figures 6C,D). Next, we applied 30 μM 8-bromo-cGMP, a cell permeable analog of cGMP to cerebellar slices in the absence of exogenous TRH. This concentration of 8-bromo-cGMP did not affect PF EPSC amplitude (Figures 7A,B). Conjunctive stimulation was then applied to induce LTD, resulting in robust LTD without application of exogenous TRH (Figures 7C,D, KO + 8-bromo-cGMP: 78.9 ± 9.0%, *P* = 0.048). These results suggest the involvement of the NO-cGMP pathway in the TRH-mediated rescue of cerebellar LTD in TRH-KO mice.

### No Significant Alteration in sIPSCs and mIPSCs in TRH-KO Mice

Previous studies have shown that TRH increased the frequency of GABA_A_ receptor-mediated sIPSCs without affecting mIPSCs in the hippocampus (Deng et al., 2006) and lateral hypothalamus (Zhang and Van Den Pol, 2012). Since TRH receptors are expressed in molecular layer interneurons (Heuer et al., 2000), inhibitory synaptic transmission from molecular layer interneurons to PCs may be altered in TRH-KO mice. To test this possibility, we recorded sIPSCs and mIPSCs from PCs, and compared the amplitudes and frequencies between KO mice and their WT littermates. However, there were no significant differences in both the amplitudes (*P* = 0.437) and frequencies (*P* = 0.893) of sIPSCs between the genotypes (*n* = 3 mice, 9 PCs in each group) (Supplementary Figures S2A–C). Next, we recorded mIPSCs after perfusing TTX. Again, there were no significant differences in both the amplitudes (*P* = 0.687) and frequencies (*P* = 0.966) of mIPSCs between the genotypes (*n* = 3 mice, 7 PCs in each group) (Supplementary Figures S2D–F).

## Discussion

Here, we report that TRH-KO mice have cerebellar LTD and motor learning deficits, without obvious morphological changes in the cerebellum. Notably, these impairments were significantly rescued by treatment with exogenous TRH. These results suggest that TRH-KO mouse cerebella lack TRH signaling, but are structurally normal. Therefore, supplementation to replace the missing TRH reliably reconstitutes defective signaling, resulting in restoration of the aberrant phenotypes.

Thyrotropin-releasing hormone triggers cellular signaling by binding to TRH receptors (Engel and Gershengorn, 2007). There are two types of TRH receptors that have been identified so far (TRH R1 and TRH R2) (O’dowd et al., 2000; Harder et al., 2001). The cerebellar cortex has been reported to contain only TRH R2, which is expressed in granule cells and molecular layer interneurons (Heuer et al., 2000). Systemic administration of TRH was demonstrated to increase the cerebellar cGMP level (Mailman et al., 1979). Since soluble guanylyl kinase, which is required for production of cGMP, is present in PCs, areas of the brain containing TRH receptors (granule cells and molecular layer interneurons) are separate from those of cGMP production (PCs). One idea proposed to reconcile the cellular inconsistency is that NO, which is produced following TRH R2 activation, acts as an anterograde messenger.

Neuronal NO synthetase is expressed in both granule cells and molecular layer interneurons (Vincent and Kimura, 1992; Rodrigo et al., 1994; Schilling et al., 1994; Ihara et al., 2006). NO produced in these cells has been shown to diffuse into neighboring PC dendrites, where it activates soluble guanylyl cyclase, leading to production of cGMP and subsequent activation of PKG in PCs (Contestabile, 2012).

**FIGURE 8:**
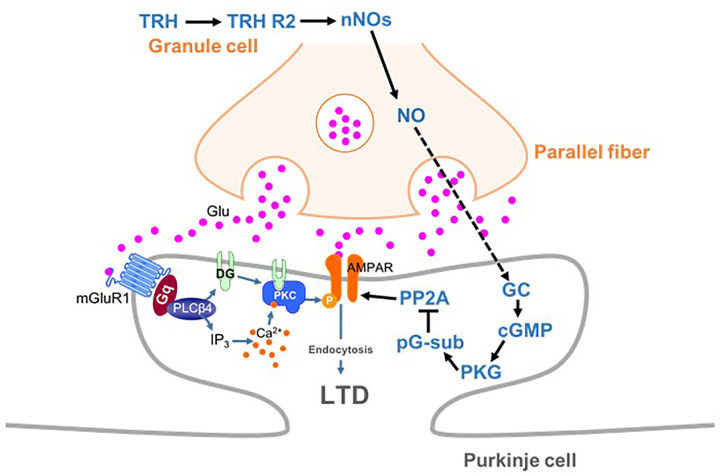
Schema depicting proposed signaling cascades regulating cerebellar LTD. AMPA receptors are phosphorylated by PKC in mGluR1-dependent manner. TRH produces NO in granule cells (and molecular layer interneurons), which diffuses into PCs. NO produces cGMP via activation of the guanylyl cyclase, leading to activation of PKG. Activated PKG phosphorylates G-substrate, which in turn suppresses PP2A. Since PP2A counteracts PKC in terms of AMPA receptor phosphorylation, suppression of PP2A facilitates LTD through enhancement of AMPA receptor phosphorylation. cGMP, cyclic guanosine monophosphate; GC, guanylyl cyclase; LTD, long-term depression; nNOs, neuronal NO synthase; pG-sub, phosphor-G-substrate; PP2A, protein phosphatase 2A; PKG, cGMP-dependent protein kinase G; TRH, thyrotropin-releasing hormone; TRH R2, TRH receptor 2.

Long-term depression at PF-PC synapses is caused by stimulation of PFs coupled with that of a CF. Since CFs convey error signals associated with inappropriate motor performance to PCs (Maekawa and Simpson, 1973), LTD is thought to contribute to motor learning by suppressing PF-PC synaptic transmissions that are related to inappropriate actions (Ito, 2001). Conjunctive stimulation for LTD induction leads to spillover of glutamate from the synaptic cleft between a PF and a PC, which in turn leads to activation of mGluR1 and PKC activation (Figure 8) (Hirai and Kano, 2018). CF-mediated depolarization leads to striking increases in the intracellular Ca^2+^ level through activation of voltage-gated Ca^2+^ channels and strengthens PKC activity. Activated PKC in turn phosphorylates the intracellular domain of the GluA2 subunit at Ser880 (Matsuda et al., 1999), leading to endocytosis of postsynaptic AMPA receptors and attenuation of synaptic strength (LTD) (Figure 8) (Matsuda et al., 2000; Hirai, 2001; Chung et al., 2003). It has been reported that NO is indispensable for cerebellar LTD (Ito and Karachot, 1990; Lev-Ram et al., 1995; Contestabile, 2012).

As discussed above, NO, which is produced by neuronal NO synthase-positive neurons (granule cells and some molecular layer interneurons), readily diffuses into PCs and activates PKG via production of cGMP (Figure 8). A previous study has proposed that G-substrate, which is abundantly and exclusively expressed in PCs, is the primary substrate for PKG (Detre et al., 1984). G-substrate phosphorylation by PKG then leads to suppression of protein phosphatase 2A (PP2A) activity (Figure 8) (Endo et al., 1999). Since PP2A dephosphorylates AMPA receptors and counteracts PKC, suppression of PP2A eventually enhances AMPA receptor phosphorylation, leading to LTD (Launey et al., 2004). Thus, NO-cGMP-PKG pathway plays a critical role in LTD by damping the PP2A activity, and consequently, facilitating AMPA receptor phosphorylation.

In conjunction with previous studies that TRH increased cGMP in the cerebellum (Mao et al., 1975; Mailman et al., 1979; Schlichter et al., 1980), we propose that TRH has a role to upregulate the basal cGMP content in PCs. In our proposed model, loss of TRH results in decreased cGMP content in PCs, and therefore, conjunctive stimulation is not sufficient to exceed a threshold of cGMP for LTD induction. Exogenous TRH or 8-bromo-cGMP compensates the loss of cGMP in KO mouse PCs, and thus, additional increase in cGMP by subsequent conjunctive stimulation can reach the threshold, and rescue LTD. However, exogenous TRH with conjunctive stimulation in the presence of L-NAME fails to upregulate cGMP content in KO PCs, because of the inhibition of NO production, resulting in failure of LTD expression.

Insufficient activation of PKC and the resultant failure of LTD has been reported in mouse models of spinocerebellar ataxia type 1 (SCA1) (Shuvaev et al., 2017) and SCA14 (Shuvaev et al., 2011). In addition, a number of previous studies have suggested that impairment of the mGluR1-PKC pathway (and aberrant PKC activation) underlie the pathologies of different types of SCAs, such as SCA3, SCA5, and SCA15/16, as reviewed recently (Hirai and Kano, 2018). It is therefore likely that treatment with TRH, which was suggested to enhance the NO-cGMP-PKG pathway in PCs, could restore cerebellar LTD by suppressing PP2A activity and eventually restoring AMPA receptor phosphorylation. In this context, it would be intriguing to investigate whether treatment with exogenous TRH or taltirelin, which is a synthetic TRH analog, could restore cerebellar LTD in mouse models of SCA demonstrated to exhibit defects in cerebellar LTD (Shuvaev et al., 2011, 2017).

In addition to LTD at PF to PC synapses, recent studies have proposed a significant contribution of two different forms of synaptic plasticity to cerebellar motor learning, long-term potentiation (LTP) at PF to PC synapses (Grasselli and Hansel, 2014; Gutierrez-Castellanos et al., 2017) and rebound potentiation (RP) at molecular layer interneurons to PC synapses (Hirano and Kawaguchi, 2014; Hirano, 2018). Since NO is also involved in the induction of postsynaptic LTP (Lev-Ram et al., 2002; Kakegawa and Yuzaki, 2005), LTP may be impaired in TRH-KO mouse cerebellum. Meanwhile, since the mild tertiary hypothyroidism was observed in the TRH-KO mice (Yamada et al., 1997), lower levels of thyroid hormone likely alter the intrinsic excitability of PCs, which could affect LTP and/or RP. Moreover, it cannot be excluded that rescue of motor learning in TRH-KO mice by exogenous TRH was attained by upregulation of thyroid hormone. Thus, considering a wide variety of physiological actions of TRH, mechanisms other than cerebellar LTD may underlie the motor learning deficit observed in TRH-KO mice. Further studies will be necessary for extensive understanding of TRH functions in the cerebellum.

## Author Contributions

MW designed and performed the experiments, analyzed the data, and drafted the manuscript. YM performed immunohistochemistry. YN, AO, and MY provided the TRH KO mice, contributed to the behavioral experiments, and discussed the study design. HH designed and supervised the experiments and wrote the manuscript.

## Conflict of Interest Statement

The authors declare that the research was conducted in the absence of any commercial or financial relationships that could be construed as a potential conflict of interest.

## Supplementary Material

Figure S1 Systemic application of TRH restores rotarod performance of TRH-KO mice to the level comparable to that of their WT littermates. **(A)** Schema showing the experimental procedure. TRH-KO and WT mice received TRH (6 ml/kg BW, 5 mg/ml) or the same volume of saline intraperitoneally (i.p.). The effects of the treatment were tested 10 min after the injection using the rotarod test. **(B)** Results of the rotarod test. Mice were subjected to four trials (*n* = 9 mice in each group). There are statistically no significant differences among four groups, as determined using repeated-measures analysis of variance. KO, knock-out; WT, wild-type.

Figure S2 No significant difference in the amplitude and frequency of both sIPSCs and mIPSCs recorded in PCs between KO mice and their WT littermates. **(A)** sIPSC traces recorded from WT mouse (left) or KO (right) mouse PCs. **(B,C)** Graphs showing averaged medial amplitudes **(B)** and inter event intervals **(C)** of sIPSCs recorded from TRH-KO mice and their WT littermates (both genotypes, *n* = 9 PCs from three mice). **(D)** mIPSC traces recorded from WT mouse (left) or KO (right) mouse PCs in the absence (upper) and presence (lower) of picrotoxin (100 μM). **(E,F)** Graphs showing averaged medial amplitudes **(E)** and inter event intervals **(F)** of mIPSCs recorded from TRH-KO mice and their WT littermates (both genotypes, *n* = 7 PCs from three mice). No statistically significant difference was detected using the Welch’s *t*-test. Scale bar: 50 pA and 1 s for **(A,D)**. KO, knock-out; mIPSCs, miniature inhibitory postsynaptic currents; N.S., not significant; PTX, picrotoxin; sIPSCs, spontaneous inhibitory postsynaptic currents; WT, wild-type.
