# Corrigendum: Contribution of thyrotropin-releasing hormone to cerebellar long-term depression and motor learning

**DOI:** 10.3389/fncel.2024.1495155

**Published:** 2024-09-26

**Authors:** Masashi Watanave, Yasunori Matsuzaki, Yasuyo Nakajima, Atsushi Ozawa, Masanobu Yamada, Hirokazu Hirai

**Affiliations:** ^1^Department of Neurophysiology and Neural Repair, Gunma University Graduate School of Medicine, Maebashi, Japan; ^2^Department of Medicine and Molecular Science, Gunma University Graduate School of Medicine, Maebashi, Japan; ^3^Research Program for Neural Signalling, Division of Endocrinology, Metabolism and Signal Research, Gunma University Initiative for Advanced Research, Maebashi, Japan

**Keywords:** thyrotropin-releasing hormone, motor learning, cerebellum, LTD, NO

In the published article, there was an error in Figure 1 as published. The captions “lobule 4/5,6” and “lobule 8,9” in the upper left panel of Figure 1A are reversed. The corrected Figure 1 appears below.

**Figure 1:**
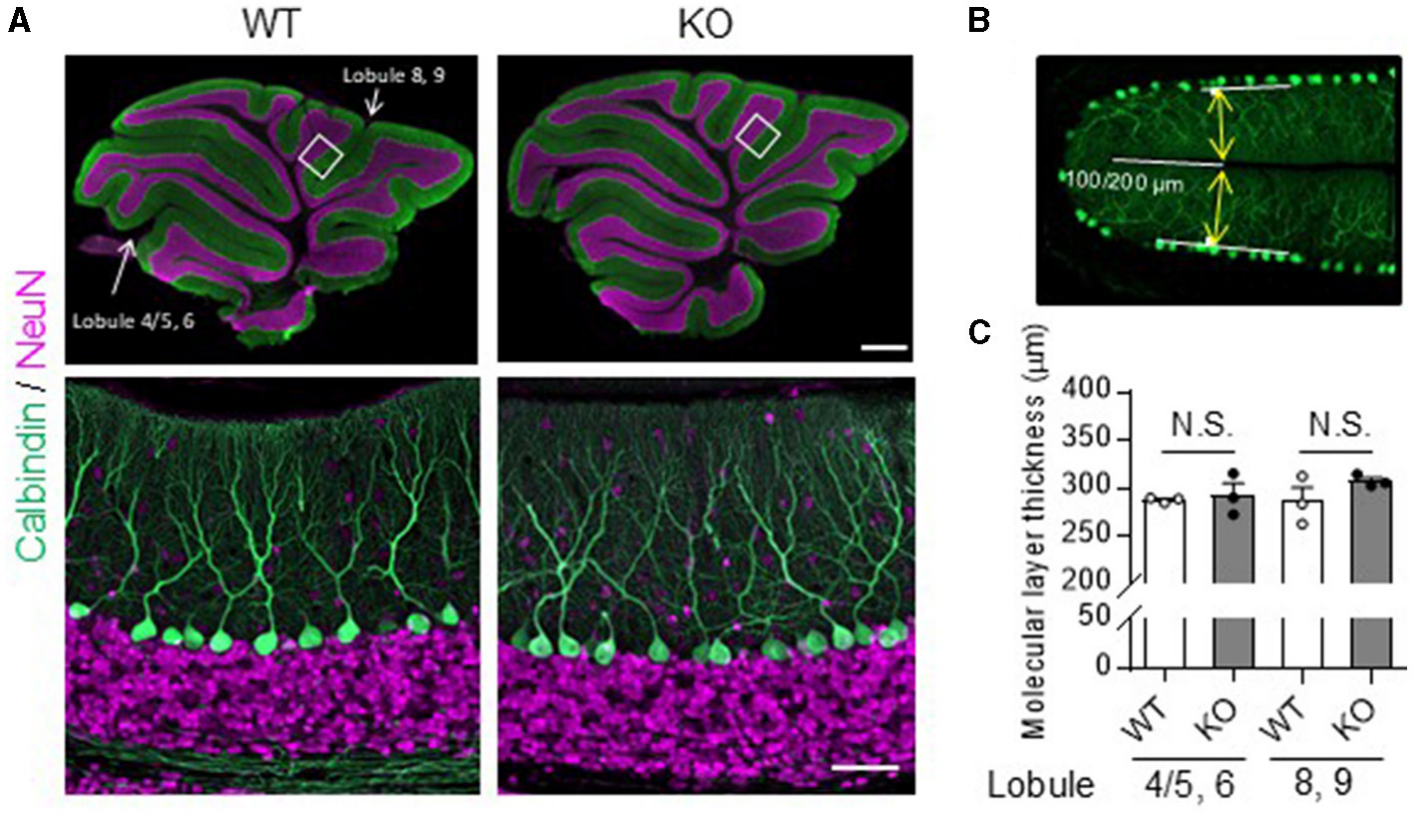
No obvious morphological differences in the cerebellum were found between TRH-KO mice and their WT littermates. Immunohistochemistry was used to compare sagittal cerebellar sections from TRH-KO mice to those from their WT littermates. Slices were double-immunostained for calbindin, a marker of PCs (green), and NeuN, a marker of granule cells (magenta). **(A)** Sagittal sections of the WT (left) and TRH-KO (right) cerebellum. The boxed areas in upper panels are enlarged. Scale bar: 500 μm (upper right) and 50 μm (lower right). KO, knock-out; WT, wild-type. **(B, C)** Quantitative analysis of the molecular layer thickness. The molecular layer thickness was measured at two different points on the sagittal section of the cerebellar vermis: lobule 4/5 and lobule 6 at 100 μm from the end of the primary fissure, and lobule 8 and lobule 9 at 200 μm from the end of the secondary fissure **(B)**. The molecular layer thickness of both sides of the fissure was measured, and the sum was defined as molecular layer thickness. There are no significant differences at both two points between genotypes **(C)**. KO, knock-out; N.S., not significant; WT, wild-type.

The authors apologize for this error and state that this does not change the scientific conclusions of the article in any way. The original article has been updated.

