# Supplementary figures and images for: Contribution of Thyrotropin-Releasing Hormone to Cerebellar Long-Term Depression and Motor Learning
Source: Front Cell Neurosci. 2018 Dec 12;12:490. doi: 10.3389/fncel.2018.00490 (PMC6299015; doi:10.3389/fncel.2018.00490)

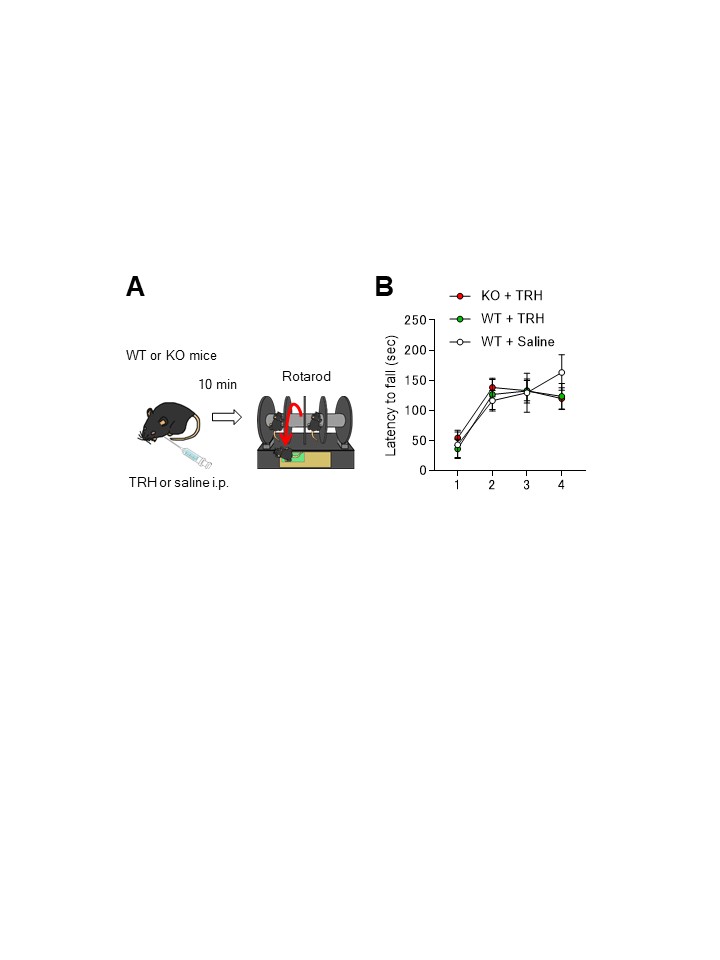

Supplement: Figure S1 — Systemic application of TRH restores rotarod performance of TRH-KO mice to the level comparable to that of their WT littermates. (A) Schema showing the experimental procedure. TRH-KO and WT mice received TRH (6 ml/kg BW, 5 mg/ml) or the same volume of saline intraperitoneally (i.p.). The effects of the treatment were tested 10 min after the injection using the rotarod test. (B) Results of the rotarod test. Mice were subjected to four trials (n = 9 mice in each group). There are statistically no significant differences among four groups, as determined using repeated-measures analysis of variance. KO, knock-out; WT, wild-type. [file Image_1.JPEG]

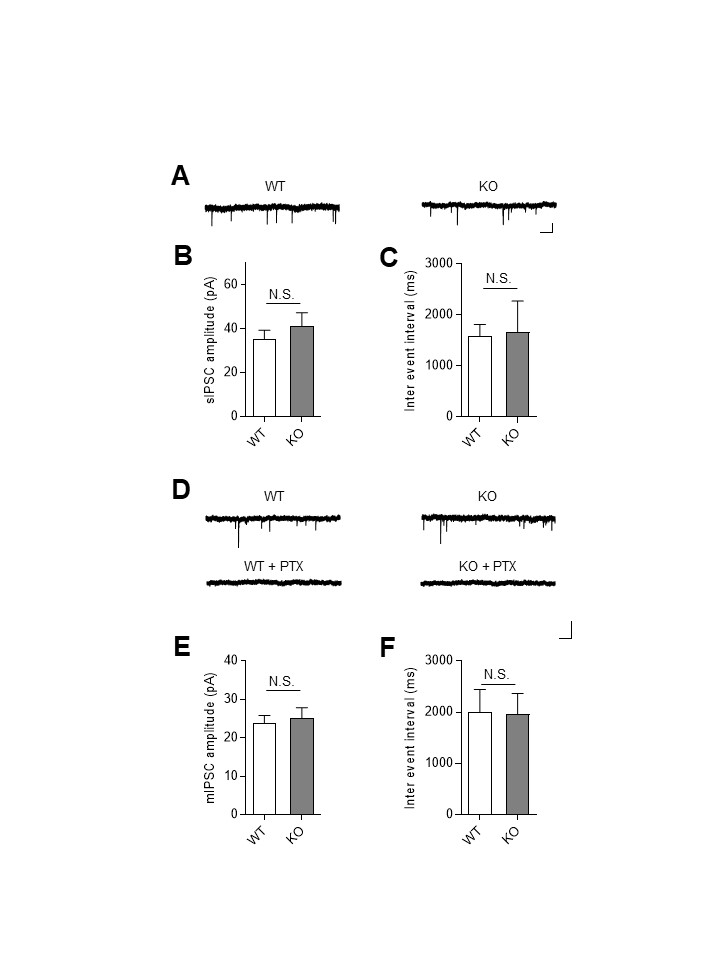

Supplement: Figure S2 — No significant difference in the amplitude and frequency of both sIPSCs and mIPSCs recorded in PCs between KO mice and their WT littermates. (A) sIPSC traces recorded from WT mouse (left) or KO (right) mouse PCs. (B,C) Graphs showing averaged medial amplitudes (B) and inter event intervals (C) of sIPSCs recorded from TRH-KO mice and their WT littermates (both genotypes, n = 9 PCs from three mice). (D) mIPSC traces recorded from WT mouse (left) or KO (right) mouse PCs in the absence (upper) and presence (lower) of picrotoxin (100 μM). (E,F) Graphs showing averaged medial amplitudes (E) and inter event intervals (F) of mIPSCs recorded from TRH-KO mice and their WT littermates (both genotypes, n = 7 PCs from three mice). No statistically significant difference was detected using the Welch’s t-test. Scale bar: 50 pA and 1 s for (A,D). KO, knock-out; mIPSCs, miniature inhibitory postsynaptic currents; N.S., not significant; PTX, picrotoxin; sIPSCs, spontaneous inhibitory postsynaptic currents; WT, wild-type. [file Image_2.JPEG]
